# Effect of *CYP3A4^∗^1G* and *CYP3A5^∗^3* Polymorphisms on Pharmacokinetics and Pharmacodynamics of Ticagrelor in Healthy Chinese Subjects

**DOI:** 10.3389/fphar.2017.00176

**Published:** 2017-03-31

**Authors:** Shuaibing Liu, Xiangfen Shi, Xin Tian, Xiaojian Zhang, Zhiyong Sun, Liyan Miao

**Affiliations:** ^1^Department of Pharmacy, The first affiliated Hospital of Zhengzhou UniversityZhengzhou, China; ^2^Department of Clinical Pharmacology Research Lab, The first affiliated Hospital of Soochow UniversitySuzhou, China

**Keywords:** ticagrelor, AR-C124910XX, *CYP3A4^∗^1G*, *CYP3A5^∗^3*, pharmacokinetics, pharmacodynamics, healthy Chinese volunteers

## Abstract

Ticagrelor is the first reversible, direct-acting, potent P2Y_12_ receptor antagonist in management of acute coronary syndromes. It is rapidly absorbed and extensively metabolized. AR-C124910XX, the major active metabolite, antagonizes the P2Y_12_ receptor at approximately equal potency. The metabolism of ticagrelor to AR-C124910XX involves CYP3A4 and CYP3A5. *CYP3A* polymorphisms have been well documented, and *CYP3A4^∗^1G* (g.20230G>A, rs2242480) and *CYP3A5^∗^3* (g.6986A>G, rs776746) are the most important single nucleotide polymorphisms in Chinese. Genetic differences in CYP3A4 and CYP3A5 expression in human volunteers and patients might affect the clearance of ticagrelor or AR-C124910XX *in vivo* resulting in subsequent variable patient response. Thus, this study is designed to explore the effects of *CYP3A4^∗^1G* and *CYP3A5^∗^3* polymorphisms on the pharmacokinetics and pharmcodynamics of ticagrelor in healthy Chinese subjects. The results indicated that the *CYP3A4^∗^1G* polymorphism significantly influenced the pharmacokinetics of AR-C124910XX, and it may be more important than *CYP3A5^∗^3* with respect to influencing ticagrelor pharmacokinetics by increasing CYP3A4 activity. However, the significant effect of *CYP3A4^∗^1G* polymorphism on AR-C124910XX plasma levels did not translate into detectable effect on inhibition of platelet aggregation. Therefore, it seems not necessary to adjust the dosage of ticagrelor according to the *CYP3A4* or *3A5* genotype.

## Introduction

Ticagrelor, a member of a novel chemical class antiplatelet agent termed cyclopentyltriazolopyrimidines, is the first reversibly binding oral adenosine diphosphate receptor antagonist acting via the P2Y_12_-receptor (Springthorpe et al., 2007; Husted and van Giezen, 2009). Compared with clopidogrel, ticagrelor significantly reduced the composite endpoint of cardiovascular death, myocardial infarction or stroke, but with similar overall major or fatal bleeding rates (Wallentin et al., 2009). Based on above advantages, it’s recommended for management of patients with acute coronary syndromes (ACS) by numerous international treatment guidelines, including the European Society of Cardiology (ESC) guidelines and the American College of Cardiology (ACC) guidelines (Wallentin et al., 2009; Levine et al., 2016).

Ticagrelor is rapidly absorbed and extensively metabolized in humans, with a total of 10 metabolites characterized by LC-MS from plasma, urine, and feces (Teng et al., 2010). Of all, only one metabolite, named AR-C124910XX, whose system exposure is approximately 30–40% of the parent compound, exhibits almost the same potency in antiplatelet effect as the parent drug (Husted et al., 2006; Butler and Teng, 2010; Teng and Butler, 2010; Teng et al., 2010; Zhou et al., 2011). Cytochrome P450 (CYP) 3A4 and 3A5 are the enzymes predominantly responsible for the metabolism of ticagrelor to AR-C124910XX (Zeng et al., 2009).

As the most abundant isoenzyme of CYP450 in the human liver, CYP3A expression varies 40-fold in individual human livers, and substrate metabolism varies at least 10-fold *in vivo* (Thummel and Wilkinson, 1998; Guengerich, 1999). It’s well documented that genetic polymorphisms contribute to 30–90% of the interindividual variability in the CYP3A activity (Evans and McLeod, 2003; Hu et al., 2006; Agrawal et al., 2010). Single nucleotide polymorphisms (SNPs) are the most common form of genetic variation in CYP3A4 and CYP3A5. Among the identified SNPs in the *CYP3A4* and *CYP3A5* genes^1^, *CYP3A4^∗^1G* (g.20230G>A, rs2242480) and *CYP3A5^∗^3* (g.6986A>G, rs776746) variants appear particularly important considering their relatively high frequency in Chinese subjects. The frequencies of *CYP3A4^∗^1G* and *CYP3A5^∗^3* alleles were 22.7–38.99% (Hu et al., 2007; Zhang et al., 2010, 2011; Yuan et al., 2011; Zhu et al., 2012; Xin et al., 2014) and 73.3% (Zhu et al., 2012; Xin et al., 2014), respectively, and *CYP3A4^∗^1G* allele is highly genetically linked with the *CYP3A5^∗^3* allele. Several studies indicate that *CYP3A4^∗^1G* is associated with altered CYP3A4 activity (Fukushima-Uesaka et al., 2004) and may be responsible for the interindividual differences in the pharmacokinetics or pharmacodynamics of tarcolimus (Shi et al., 2011; Zhu et al., 2012; Li et al., 2013, 2014), atorvastatin (Gao et al., 2008; He et al., 2014), cyclosporine (Hu et al., 2007; Qiu et al., 2008), and postoperative fentanyl requirements (Zhang et al., 2010, 2011; Liao et al., 2013); *CYP3A5^∗^3* allele is associated with a non-functional protein due to a premature termination codon. Therefore, altered activity of CYP3A4 and CYP3A5 resulting from genetic polymorphisms may affect the formation of active metabolite and the antiplatelet effect of the drug subsequently. However, as far as we know, the evidence is limited about whether *CYP3A4^∗^1G* and *CYP3A5^∗^3* polymorphisms affect the pharmacokinetics and pharmacodynamics of ticagrelor and AR-C124910XX in Chinese subjects.

The aim of our study is to explore the role of the two genetic plymorphisms in pharmacokinetics and pharmacodynamics of ticagrelor and AR-C124910XX in healthy Chinese subjects. We hope the results can provide references for future clinical individualized dosage regimens.

## Subjects and Methods

### Subjects

Fourteen healthy male Chinese volunteers who had the *CYP3A4^∗^1G* (g.20230G>A, rs2242480) or *CYP3A5^∗^3* (g.6986A>G, rs776746) variant or both were recruited from 136 male healthy Chinese volunteers. Each subject was physically healthy with no prior history of significant medical illness according to a medical history, physical examinations, vital signs, routine clinical laboratory tests (complete blood count and tests for renal and hepatic function, and coagulation) and ECGs. Subjects were required to have the following laboratory values within normal ranges: body mass index (BMI) of 19–24 kg⋅m^-2^, baseline maximal platelet aggregation (MPA) response to 5 μM ADP of ≥70%. Volunteers who had donated blood within the past 2 months, who had a history of conditions affecting drug disposition (e.g., allergies, genetic diseases); who had a personal or family history of bleeding disorders/events; who smoked ≥5 cigarettes per week (or the equivalent use of other nicotine-containing products); who consumed grapefruit products 1 week before the study, or who had taken any prescription medication within 2 weeks of the start of the study were excluded.

All subjects provided written informed consent before participation and the Ethics Committee of the First Affiliated Hospital of Soochow University approved the study protocol. Studies were conducted in accordance with the International Conference on Harmonisation Guideline for Good Clinical Practice and the Declaration of Helsinki.

### Genotyping

Genomic DNA from EDTA-treated blood samples was extracted using a Wizard Genomic DNA Purification kit (Promega Corporation, Madison, WI, USA) according to the manufacturer’s instructions. Genomic DNA extracted from subjects’ blood was genotyped for *CYP3A4^∗^1G* or *CYP3A5^∗^3* alleles by direct sequencing. Briefly, the genomic DNA was amplified by use of two primers for each genotype. The sequences of PCR primers are shown in **Table 1**. PCR was performed by using Taq DNA polymerase (1.25 U/50 μL), 10× Taq Buffer, 25 mM MgCl_2_, dNTP and genomic DNA (50–100 ng/50 μL) with a pair of primers (0.3 μM) on T100^TM^ Thermal Cycler. The PCR conditions for *CYP3A4^∗^1G* were 95°C for 5 min, followed by 35 cycles of denaturizing at 95°C for 30 s, annealing at 59°C for 30 s, and extension at 72°C for 30 s, and a final extension at 72°C for 10 min. The PCR conditions for *CYP3A5^∗^3* is almost the same as *CYP3A4^∗^1G* except for the annealing temperature that was 56°C. Then, the genotyping was carried out by direct sequencing on an ABI 3730 DNA Analyzer.

**Table 1:** The sequences of PCR primers for genotyping.

SNP	Primers	
*CYP3A4^∗^1G*	Forward:	5′-CACCCTGATGTCCAGCAGAAACT-3′
	Reverse:	5′-AATAGAAAGCAGATGAACCAGAGCC-3′
*CYP3A5^∗^3*	Forward:	5′-CATGACTTAGTAGACAGATGA-3′
	Reverse:	5′-GGTCCAAACAGGGAAGAAATA-3′

### Study Protocol

All subjects were admitted to the clinical trial center the night before ticagrelor dosing. At 7:00 AM on the subsequent morning, each subject received one dose (180 mg, *po*) with 200 mL of water after an overnight fast. Blood samples for pharmacokinetics (4 mL each) were collected via an indwelling catheter or direct venipuncture into tubes containing sodium heparin immediately before and 0.5, 1, 1.5, 2, 3, 4, 5, 6, 8, 12, 16, 24, 36, and 48 h after drug administration. Blood samples were processed by centrifugation (4,000 rpm at 4°C for 5 min), and plasma was harvested and stored at -80°C until analysis. Pharmacodynamics samples were collected via direct venipuncture into tubes containing 0.109 mmol⋅L^-1^ sodium citrate before and 1, 2, 4, 12, 24, and 48 h after drug administration.

### Pharmacokinetic Assessments

Plasma concentrations of ticagrelor and the metabolite, AR-C124910XX, were analyzed by validated UPLC-MS-MS methods as previously described (Sillen et al., 2010). Protein in plasma samples was precipitated with acetonitrile, and samples were chromatographed using a Waters ACQUITY UPLC BEH C18 (2.1 × 100 mm, 1.7 μm) column with a mobile phase consisting of acetonitrile and 0.1% formic acid water (55: 45, v/v) at a flow rate of 300 μL⋅min^-1^. The linear calibration ranges were 0.5–2,000 ng⋅mL^-1^ for ticagrelor and AR-C124910XX (*r*^2^ ≥ 0.99) and the lower limits of quantification for ticagrelor and AR-C124910XX were both 0.5 ng⋅mL^-1^. Intraday precision values [relative standard deviations (RSD)] ranged from 6.0 to 13.2% for ticagrelor and 4.2–11.5% for AR-C124910XX at three quality control levels. Interday precision values ranged from 8.7 to 11.4% for ticagrelor and 6.5 to 10.2% for AR-C124910XX.

### Pharmacodynamics Assessments

The inhibition of ADP-induced platelet aggregation of platelet-rich plasma (PRP) was measured in response to 5 μM ADP as described elsewhere (Muller et al., 2003; Geiger et al., 2005). The measurement was achieved on a platelet aggregation profiler-4 optical aggregometer with temperature maintained at 37°C. The observed maximal platelet aggregation was recorded, and the effects on ADP-induced inhibition of platelet aggregation (IPA; the primary pharmacodynamics parameter) were calculated using the following equation:

(1) $${\text{IPA}}_{\text{t}}\;(\%\;\text{inhibition})\;=\;\left[\frac{\left({\text{MPA}}_{\text{baseline}}\;-\;{\text{MPA}}_{\text{t}}\right)}{{\text{MPA}}_{\text{baseline}}}\right]\;\times \;100\%,$$

where IPA_t_ represents the IPA at time *t*, MPA_t_ represents the MPA at time *t*, and MPA_baseline_ represents the MPA value at baseline. In essence, a higher IPA indicated greater antiplatelet effect.

### Pharmacokinetic and Pharmacodynamics Analysis

Pharmacokinetics was analyzed using non-compartmental methods and WinNonlin Pro 5.2 (Pharsight Corporation, Mountain View, CA, USA). Concentration–time curves were generated for plasma ticagrelor and AR-C124910XX, and C_max_ was estimated directly from observed plasma concentration–time data. AUC_0-t_ was calculated using the linear trapezoidal rule, AUC_0-∞_ was calculated as AUC_0-∞_ = AUC_0-48_ + C_t_/k_e_, where C_t_ was the last measured concentration and k_e_ was calculated using linear regression analysis of the log-linear part of the plasma concentration–time curve. t_1/2_ was calculated as ln2/k_e_.

The pharmacodynamics effect (IPA) of ticagrelor was expressed as the percentage change from baseline platelet aggregation to 48 h after initial administration of the study drugs. The area under the time-effect curve (AUEC) for the IPA of ticagrelor was calculated from the time vs. IPA value curve, using the linear trapezoidal rule.

## Statistical Analysis

Statistical analyses were performed using SPSS 16.0. Continuous variables were presented as means ± SD, and a Kolmogorov–Smirnov test was used to confirm normal distribution of continuous data. Data normally distributed were analyzed using a one-way analysis of variance (ANOVA) with the least significant difference (LSD) *post hoc* test for multiple comparisons and an unpaired Student’s *t*-test for two groups as appropriate. Comparison of data not normally distributed was performed with a Mann–Whitney *U* test for comparison of two groups, while a Kruskal–Wallis *H* test was used for comparison of multiple groups. Two-sided *P* values ≤ 0.05 were considered statistically significant.

### Tolerability

All participants given the medication evaluated here were observed closely to preserve their safety. All adverse events (AEs), such as symptoms and their severity, duration, time of onset and disappearance, and relationships to the study drug, were recorded. Physical examinations were performed and vital signs were evaluated, including systolic and diastolic blood pressures. Laboratory tests included urinalysis and hematologic, coagulation, and chemical analysis were performed.

## Results

### Genotyping

Fourteen healthy Chinese male subjects were recruited from 136 individuals genotyped for *CYP3A4^∗^1G* and *CYP3A5^∗^3*. All enrolled subjects completed the study with no major protocol violations. Subjects’ demographics and SNP characteristics appear in **Table 2**, and there were no significant differences in age, weight, height, or BMI among the six genotypes.

**Table 2:** Demographics of 14 healthy Chinese male volunteers according to *CYP3A4* and *CYP3A5* genotypes; values are mean (*SD*).

SNP	No.	Age, y	Weight, kg	Height, cm	Body mass index, kg/m^2^
*CYP3A4^∗^1/^∗^1*	6	26.2 (2.5)	62.5 (3.9)	171.2 (3.7)	21.3 (1.4)
*CYP3A4^∗^1/^∗^1G*	6	26.7 (2.8)	63.3 (5.3)	170.2 (6.7)	21.9 (1.9)
*CYP3A4^∗^1G/^∗^1G*	2	25.0 (0)	59.5 (0.7)	171.0 (5.7)	20.4 (1.6)
*CYP3A5^∗^1/^∗^1*	3	27.7 (2.5)	63.0 (3.6)	166.0 (2.6)	22.9 (1.2)
*CYP3A5^∗^1/^∗^3*	4	25.8 (2.9)	59.4 (3.4)	172.0 (2.6)	20.1 (1.1)
*CYP3A5^∗^3/^∗^3*	7	25.9 (2.2)	63.9 (4.6)	172.0 (5.9)	21.6 (1.6)

### Association of *CYP3A4^∗^1G* and *CYP3A5^∗^3* Polymorphisms with Ticagrelor and AR-C124910XX Pharmacokinetics

**FIGURE 1:**
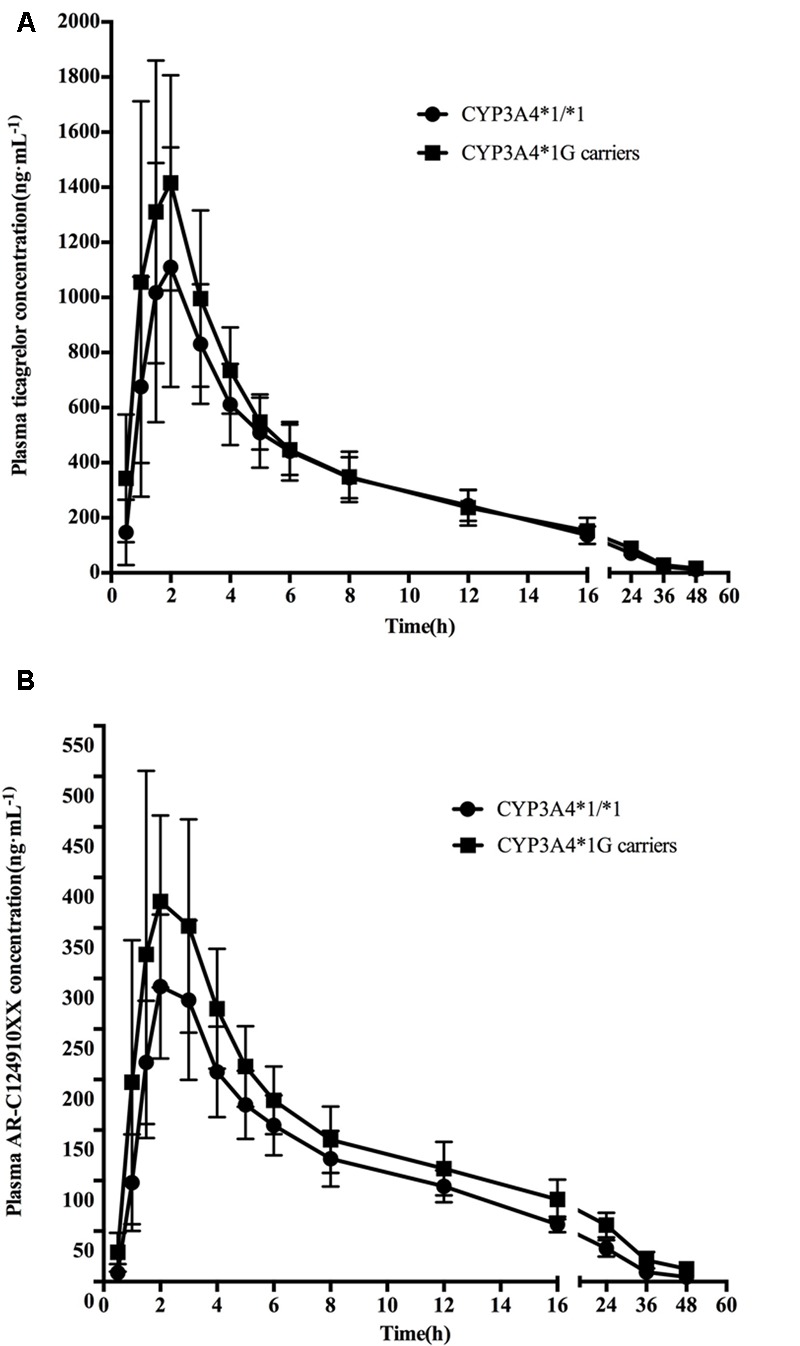
**Mean (SD) plasma concentration–time profiles of ticagrelor (A)** and AR-C124910XX **(B)** after administration of single 180 mg oral dose for the different *CYP3A4^∗^1G* genotypes.

**FIGURE 2:**
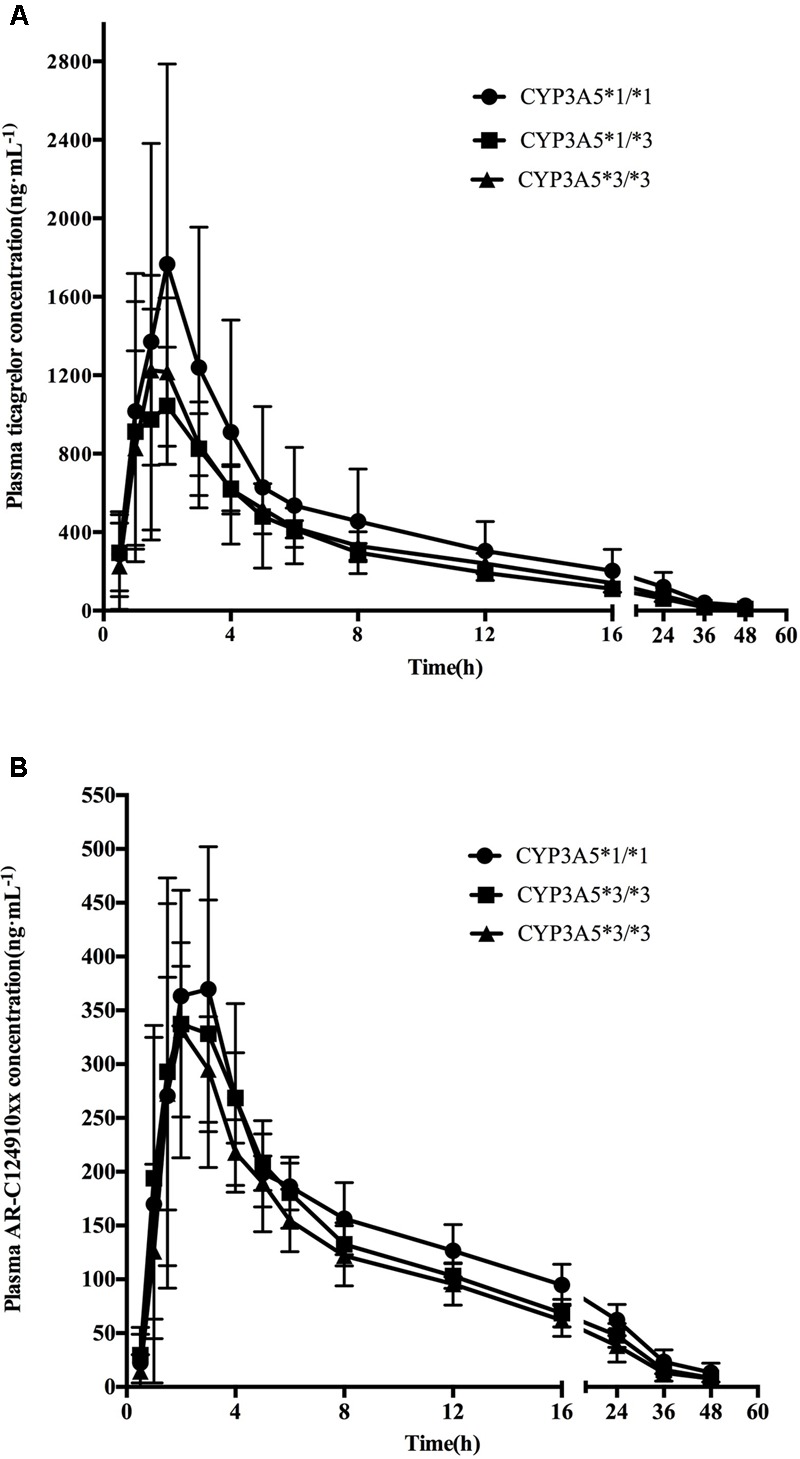
**Mean (SD) plasma concentration–time profiles of ticagrelor (A)** and AR-C124910XX **(B)** after administration of single 180 mg oral dose for the different *CYP3A5^∗^3* genotypes.

Mean ± SD plasma concentration–time profiles for ticagrelor and AR-C124910XX for *CYP3A4* and *CYP3A5* genotypes are presented in **Figures 1, 2**. Pharmacokinetic comparisons for both genotypes are summarized in **Tables 3, 4**. Because no significant differences exist between the CYP3A4: g.20230GA heterozygotes and CYP3A4: g.20230AA homozygotes, both were combined and compared to CYP3A4: g.20230GG homozygotes.

**Table 3:** Pharmacokinetic properties of ticagrelor and AR-C124910XX after administration of single 180 mg oral dose for the different *CYP3A4^∗^1G* genotypes; values are as mean (*SD*).

Pharmacokinetic parameters	Ticagrelor		AR-C124910XX	
	*CYP3A4^∗^1/^∗^1*	*CYP3A4^∗^1G carriers*	*CYP3A4^∗^1/^∗^1*	*CYP3A4^∗^1G carriers*
	*n* = 6	*n* = 8	*n* = 6	*n* = 8
AUC_0-last_(ng mL^-1^ h^-1^)	8241.8 (1908.9)	9608.9 (4065.5)	2828.7 (329.1)^∗∗^	3907.1 (569.5)
AUC_0-∞_(ng mL^-1^ h^-1^)	8379.0 (1943.7)	9831.03 (4268.0)	2887.7 (347.7)^∗∗^	4124.4 (659.3)
t_1/2_(h)	7.9 (1.0)	8.6 (1.2)	7.9 (1.8)^∗∗^	11.0 (2.2)
t_max_(h)	2.0 (0.6)	1.8 (0.6)	2.1 (0.5)	2.2 (0.7)
C_max_(ng mL^-1^)	1225.3 (421.70)	1613.4 (570.2)	310.6 (53.6)^∗∗^	440.0 (69.8)

*^∗∗^*P* < 0.01*

**Table 4:** Pharmacokinetics properties of ticagrelor and AR-C124910XX after administration of single 180 mg oral dose for the different *CYP3A5^∗^3* genotypes; values are mean (*SD*).

Pharmacokinetic parameters	Ticagrelor			AR-C124910XX		
	*CYP3A5^∗^1/^∗^1*	*CYP3A5^∗^1/^∗^3*	*CYP3A5^∗^3/^∗^3*	*CYP3A5^∗^1/^∗^1*	*CYP3A5^∗^1/^∗^3*	*CYP3A5^∗^3/^∗^3*
	*n* = 3	*n* = 4	*n* = 7	*n* = 3	*n* = 4	*n* = 7
AUC_0-last_(ng mL^-1^⋅h^-1^)	11884.0 (6564.2)	7615.4 (941.4)	8601.3 (1720.5)	4144.9 (544.7)	3527.3 (747.2)	3098.0 (604.4)
AUC_0-∞_ (ng mL^-1^⋅h^-1^)	12236.1 (6879.7)	7715.7 (948.7)	8764.4 (1754.6)	4375.2 (681.0)	3658.3 (807.4)	3223.2 (730.0)
t_1/2_(h)	9.0 (1.7)	7.6 (0.5)	8.3 (1.1)	10.7 (3.0)	9.8 (2.1)	9.2 (2.8)
t_max_(h)	1.7 (0.6)	2.0 (0.8)	1.9 (0.6)	2.2 (0.8)	2.4 (0.8)	2.0 (0.5)
C_max_(ng mL^-1^)	1926.7 (773.7)	1181.9 (479.5)	1393.0 (365.7)	440.3 (97.0)	395.8 (120.5)	354.2 (67.3)

### Ticagrelor

No statistically significant differences were observed in the AUC_0-last_, AUC_0-∞_, t_1/2_, t_max_, or C_max_ between the two *CYP3A4* genotypes (*P* > 0.05). No statistically significant differences were found in pharmacokinetics for ticagrelor among the three *CYP3A5* genotypes.

### AR-C124910XX

The mean AUC_0-last_, AUC_0-∞_, and C_max_ of AR-C124910XX in *CYP3A4^∗^1G* carriers were 1.38 (*P* = 0.001), 1.43 (*P* = 0.001), and 1.42 (*P* = 0.003)-fold higher than in those with the CYP3A4: g.20230GG homozygotes, respectively. The t_1/2_ of AR-C124910XX in the *CYP3A4^∗^1G* group was also significantly longer than the CYP3A4: g.20230GG homozygotes (*P* = 0.015). The *CYP3A4* genotype had no statistically significant effect on the t_max_ of AR-C124910XX. The *CYP3A5* genotype had no statistically significant effect on pharmacokinetics parameters of AR-C124910XX.

### Association of *CYP3A4^∗^1G* and *CYP3A5^∗^3* Polymorphisms with Ticagrelor and AR-C124910XX Pharmacodynamics

**FIGURE 3:**
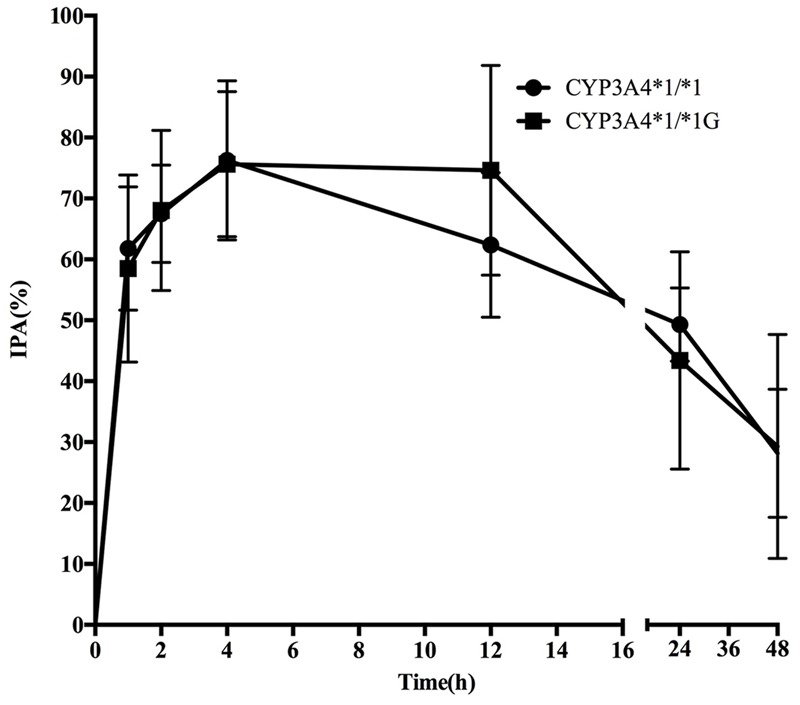
**Mean (SD) inhibition of platelet aggregation (IPA,%) of ticagrelor after administration of single 180 mg oral dose for the different *CYP3A4^∗^1G* genotypes**.

**FIGURE 4:**
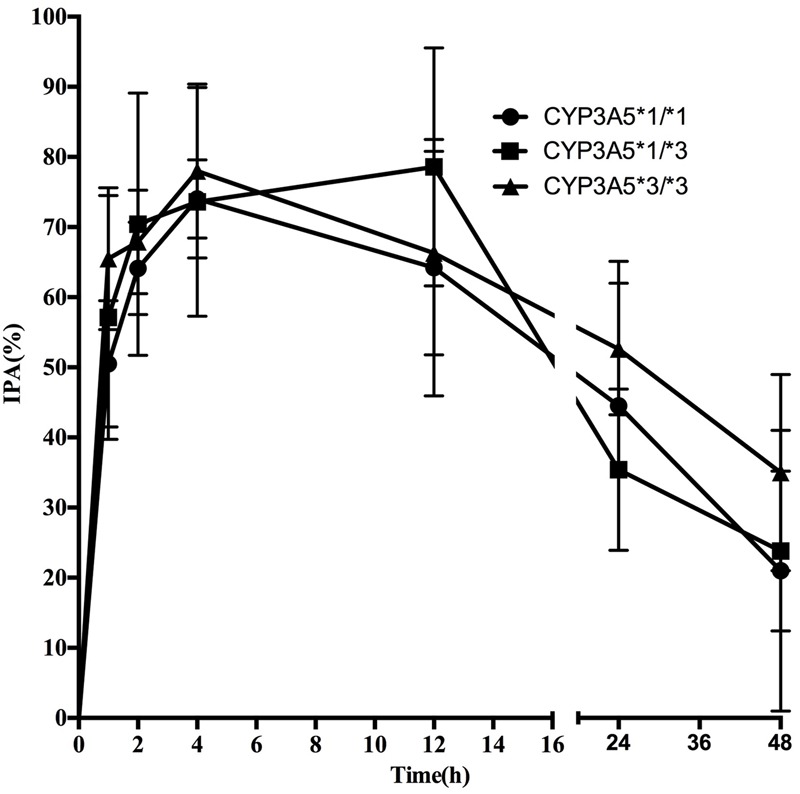
**Mean (SD) IPA(%) of ticagrelor after administration of single 180 mg oral dose for the different *CYP3A5^∗^3* genotypes**.

Pharmacodynamics properties of ticagrelor for *CYP3A4* and *CYP3A5* genotypes are presented in **Figures 3, 4**. Pharmacodynamics comparisons for both genotypes are summarized in **Table 5**. Strong inhibitory effects on platelet aggregation were observed. A single oral dose of ticagrelor (180 mg) induced a maximum inhibition of 75% at an average of 4 h after dosing. No statistically significant difference in IPA was observed for either *CYP3A4* or *CYP3A5* genotype.

**Table 5:** Inhibition of platelet aggregation [area-under-the-effect curve (AUEC), % inhibition⋅h] over time of ticagrelor after administration of single 180 mg oral dose for the different *CYP3A4* and *CYP3A5* genotypes.

Pharmacodynamic parameter	*CYP3A4^∗^1/^∗^1*	*CYP3A4^∗^1G carriers*	*CYP3A5^∗^1/^∗^1*	*CYP3A5^∗^1/^∗^3*	*CYP3A5^∗^3/^∗^3*
	*n* = 6	*n* = 8	*n* = 3	*n* = 4	*n* = 7
AUEC (%inhibition⋅h)	2393.33 (283.86)	2417.88 (614.15)	2212.00 (686.93)	2239.75 (366.86)	2586.86 (455.58)

*Values are represented as mean (SD).*

### Tolerability

Ticagrelor was well tolerated, and no study volunteers withdrew from the protocol, and no clinically significant bleeding event or thrombocytopenia was documented.

## Discussion

Effective antiplatelet treatment is particularly important in patients with a high risk of cardiovascular events. As an orally active antiplatelet agent, ticagrelor has been demonstrated to exert a faster and more powerful IPA in comparison to clopidogrel in coronary artery disease patients. Although ticagrelor does not require metabolic activation for antiplatelet activity, it is still extensively metabolized to an active metabolite, AR-C124910XX (Teng et al., 2010; Giorgi et al., 2011). Significantly altered plasma concentration of ticagrelor or AR-C124910XX has been reported after the concomitant use of ketoconazole (CYP3A4 inhibitor) or rifampicin (CYP3A4 inducer). Therefore, it is plausible that modified CYP3A4 and 3A5 enzyme activity caused by genetic polymorphisms may potentially influence the exposure of the parent drug or metabolite *in vivo* resulting in subsequent variable patient response. However, influence of *CYP3A4^∗^1G* and *CYP3A5^∗^3* on ticagrelor pharmacokinetics and pharmacodynamics is still not quite clear. The present study was thus designed to extensively investigate the effects of *CYP3A4^∗^1G* and *CYP3A5^∗^3* genetic polymorphisms on the pharmacokinetics and pharmacodynamics of ticagrelor and AR-C124910XX in Chinese healthy subjects.

Indeed, our results suggest that subjects with the *CYP3A4^∗^1G* allele had greater AR-C124910XX AUC_0-last_, AUC_0-∞_, and C_max_ and longer t_1/2_ compared with those subjects with the *CYP3A4^∗^1/^∗^1* genotypes. Thus, the *CYP3A4^∗^1G* allele may enhance CYP3A4 expression and increase biotransformation of ticagrelor. This finding is consistent with previously published studies (Hu et al., 2007; Qiu et al., 2008; Zeng et al., 2009) which showed that the *CYP3A4^∗^1G* genotype significantly affects cyclosporine pharmacokinetics and increases its CYP-mediated metabolism. In contrast, Zhang and Qin’s groups (Zhang et al., 2010; Liao et al., 2013) found that *CYP3A4^∗^18B* (*CYP3A4^∗^1G*) polymorphism decreased CYP3A4 activity and modified fentanyl pharmacokinetics. So, patients with the *CYP3A4^∗^1G/^∗^1G* genotype required significantly less fentanyl for postoperative pain control compared to those with wild type or the *CYP3A4^∗^1/^∗^1G* genotype.

However, statistically significant difference in IPA was not observed as well as the effects of *CYP3A4^∗^1G* on plasma AR-C124910XX levels. Considering the fact that CYP3A4 and CYP3A5 convert ticagrelor into its active metabolite, AR-C124910XX, whose potency is almost the same as ticagrelor in antagonizing P2Y_12_ receptor, it became not so surprised. However, it’s important to note that a strong linkage disequilibrium exists between the *CYP3A4^∗^1G* and *CYP3A5^∗^1* polymorphisms, the presence of ^∗^1G might be only a marker of yet unstudied allele of greater influence of the variability on ticagrelor therapy. Therefore, to further examine the associations of the *CYP3A5^∗^3* and *CYP3A4^∗^1G* polymorphisms on CYP3A activity and to assess the combined influence of these two polymorphisms, a larger study should be on the way.

Varenhorst et al. (2015) reported GWAS data regarding ticagrelor pharmacokinetics in a large cohort of ticagrelor-treated ACS patients and showed an association of three different genetic loci (*SLCO1B1, CYP3A4*, and *UGT2B7*) with plasma ticagrelor. Because the *CYP3A4*:(g.11107G>A, rs56324128) is infrequent in the Chinese, we selected *CYP3A4^∗^1G* for the study, and our data showed that *CYP3A4^∗^1G* significantly affected AR-C124910XX pharmacokinetics but not the parent drug or platelet reactivity, which is in agreement with data published by Christophe’s group. Meanwhile, considering the fact that, there was no direct evidence whether SLCO1B1 or UGT2B7 participated in the metabolism of ticagrelor, and the effect of genetic polymorphisms of the two proteins on ticagrelor weren’t assessed in our study.

## Conclusion

In summary, the *CYP3A4^∗^1G* polymorphism significantly influenced the pharmacokinetics of AR-C124910XX, and it may be more important than *CYP3A5^∗^3* with respect to influencing ticagrelor pharmacokinetics by increasing CYP3A4 activity. Dosage adjustment according to the *CYP3A4* or *3A5* genotype seems unnecessary considering their minor impact on platelet reactivity.

## Author Contributions

SL, LM, and ZS contributed to the study design and protocol. SL and XS conducted the study. SL, XZ, and XT performed the pharmacokinetic and pharmacodynamics measurements. All authors reviewed, interpreted the data and agreed on the content. LM and ZS were the lead authors, directed the manuscript content of each draft, supervising the medical writer. All authors approved the final version for submission.

## Conflict of Interest Statement

The authors declare that the research was conducted in the absence of any commercial or financial relationships that could be construed as a potential conflict of interest.

## Footnotes

1 http://www.cypalleles.ki.se/
